# Association of Mental Health Disorders and Social Determinants of Health with Frequent Emergency Department Use

**DOI:** 10.5811/westjem.35599

**Published:** 2025-07-18

**Authors:** Derick D. Jones, Luis Santos Molina, Aidan Mullan, Ronna L. Campbell

**Affiliations:** * Mayo Clinic, Department of Emergency Medicine, Rochester Minnesota; † Mayo Clinic, Department of Quantitative Health Sciences, Rochester, Minnesota

## Abstract

**Introduction:**

Patients who frequently use the emergency department (ED) make up 8% of ED patients annually but account for up to 28% of all ED visits. Frequent ED utilization has been associated with mental health disorders. However, the association between social determinants of health (SDoH) and frequent ED use is not as well understood. Our objective was to identify associations between frequent ED use and mental health disorders and SDoH among patients visiting 19 Upper Midwest EDs in an integrated health system.

**Methods:**

We conducted a cross-sectional analysis of adult patients presenting to the 19 EDs from July 1, 2020–June 30, 2021. Using odds ratios (OR) and 95% confidence intervals obtained from multivariable logistic regression models, we characterized associations between mental health disorders (based on ICD-10 groupings) and 10 SDoH with frequent ED utilization (defined as ≥6 ED visits per year).

**Results:**

A total of 228,814 visits among 134,452 patients were eligible for inclusion. After accounting for clinical features and mental health risk factors, the following had the strongest association with frequent ED use: unmet transportation needs (OR 1.73); high risk for financial resources (OR 1.52); food insecurity (OR 1.58); smoking tobacco (OR 1.31); and physical inactivity (OR 1.23). The top mental health risk factors for frequent ED utilization were adult personality and behavioral disorders (OR 4.0) and anxiety, stress and non-psychotic disorders (OR 3.35).

**Conclusion:**

We found strong associations between mental illness and SDoH and frequent ED use. The strongest SDoH risk factors included unmet transportation needs, financial resource risk, and food insecurity. The top two mental health risk factors were adult personality and behavioral disorders as well as anxiety and stress disorders, with differences that persisted when analyzed independently as well as when adjusting for other mental health risk factors. By understanding the interaction between social determinants of health and mental health disorders researchers can better address root causes and improve health outcomes among this vulnerable population.

## INTRODUCTION

Patients who frequently use the emergency department (ED) comprise up to 8% of all individuals visiting the ED, but they account for up to 28% of visits.^1^–^4^ These patients tend to suffer from high rates of psychiatric illness,^5^–^7^ are more likely to be undomiciled,^4^,^8^–^11^ have public insurance or lack of insurance,^12^,^13^ suffer from substance use disorders,^1^,^2^,^4^,^5^,^7^,^14^–^17^ and have higher risk of mortality and hospital admission.^18^ Addressing community social determinants of health (SDoH) is increasingly being recognized as a key factor in healthcare utilization, with patients who live in under-resourced neighborhoods having 55% more encounters in the health system as compared to those who are in well-resourced neighborhoods.^19^ In this study we aimed to identify the specific mental health and social needs factors that contribute to ED use.

### Knowledge Gaps

Although prior studies have demonstrated increased rates of psychiatric illness among patients who frequently use healthcare resources,^1^,^5^–^8^,^13^–^15^ these studies lack a standardized approach using diagnostic code groupings to better understand which categories of psychiatry illness carry the most risk. Furthermore, although SDoH are increasingly recognized as predictors of healthcare disparities, reflecting the economic, political, social, and physical environments that impact access to healthcare and disproportionality in disease burden, there is a paucity of research on the impact of SDoH in combination with psychiatric illness on frequent ED use.^20^,^21^

### Study Objectives

We hypothesized an association between SDoH, psychiatric illness, and frequent ED use that can provide insight into how best to meet the underlying needs of this vulnerable population. Our objective in this study was to assess for associations of each of these risk factors with frequent ED use defined as ≥ 6 visits in a calendar year. We sought to assess the interactions between psychiatric illness and SDoH to highlight additional risk factors for frequent ED utilization beyond the previously studied psychiatric illness risk factors.

## METHODS

### Study Design and Setting

We conducted a multicenter, cross-sectional analysis of 19 EDs within an integrated health system in the Upper Midwest. The ED visits were identified from 19 Mayo Clinic EDs in Minnesota and Wisconsin from July 1, 2020 – June 30, 2021. These EDs encompass a regional health system with practice locations ranging from small, rural hospitals in southern Minnesota and western Wisconsin to an academic, quaternary-care hospital in Rochester, MN. The population selection included all ED encounters during this time frame.

Visits were excluded if the patient did not provide research authorization or was <18 years of age. Our primary outcome of interest was ED use calculated as the number of visits by the same patient in the previous 12 months. Thresholds for utilization were determined as ≥ 3 visits, ≥ 6 visits, or ≥ 18 visits in the prior year. Frequent ED use is defined in different ways in the literature; however, the most commonly reported threshold is six visits, which we chose for our primary analysis. The study adheres to the STROBE (Strengthening the Reporting of Observational Studies in Epidemiology) and RECORD (Reporting of Studies Conducted Using Observational Routinely-collected Data) reporting guidelines and was exempted from review by the Mayo Clinic Institutional Review Board.^22^,^23^

**Population Health Research Capsule**

What do we already know about this issue?

*Associations between social determinants of health (SDOH) and mental health disorders for patients who frequently use the Emergency Department (ED) is not well understood*.

What was the research question?

*Identify associations between frequent ED use and mental health disorders and SDOH and frequent ED utilization*.

What was the major finding of the study?

*Top risk factors: transport (OR 1.7), finances (OR 1.5), food insecurity (OR 1.6), personality disorders (OR 4.0), anxiety (OR 3.4), p<0.001*.

How does this improve population health?

*By understanding the interactions between social determinants of health and mental health disorders researchers can address root causes and improve health outcomes for this population*.

### Variables

Primary variables of interest were patient responses to SDoH surveys and active mental health diagnoses. The SDoH survey responses were collected using an institutional survey methodology administered annually on an outpatient basis for patients at ambulatory clinic appointments. Active mental health diagnoses were provided by primary care and mental healthcare teams for which the patients were enrolled. Only a portion of ED patients had data from SDoH surveys due to survey methodology, as well as gaps in enrollment with ambulatory care teams. Components of the SDoH survey were collected via social history across healthcare encounters by registration staff, nurses, or clinicians. To address response bias we evaluated the SDoH risk factors individually rather than excluding patient visits with partial data availability, and the responses to SDoH items were grouped for analysis.

Active mental health diagnoses were identified through the patient “active problems” list. Mental health diagnoses were categorized based on the *International Classification of Diseases, Rev 10* (ICD-10) codes recorded as active problems at the time of ED visit and flagged as present or not-present regardless of the number of distinct diagnoses from a single category. Other variables of interest included patient demographics, age- and disease severity-weighted Charlson Comorbidity Index (CCI) score, and insurance type. Only comorbid diagnoses occurring within three years of the ED visit were included in the CCI score. Insurance was classified as Medicare, Medicaid, private, self-pay, or other. We also assessed patient primary language, means of arrival, arrival day of the week and time of day, chief concern, triage Emergency Severity Index (ESI) score, modified early warning score with a 24-hour window (MEWS24), primary ED diagnosis, and ED disposition.^24^,^25^ Categorization of patient chief concerns and ED have been previously described.^26^

### Statistical Methods

We summarized patient demographics and visit characteristics across ED use groups using means with standard deviations and medians with interquartile ranges for continuous features, or frequency counts and percentages for categorical measures. Associations between patient risk factors and high ED utilization were assessed using population-averaged logistic generalized estimating equations (GEE). The benefit to using GEEs over standard logistic regression is that GEEs account for multiple ED visits from a single patient during the study period, which cannot be assumed to be independent from one another. Moreover, by analyzing across all ED visits we allowed individual patients to change ED use status and update their SDoH survey results during the study period. For these analyses, we assumed that visits from individual patients had constant correlation; we calculated covariance using the Eicker-Huber-White estimator.

We constructed three sets of GEE models for this analysis. First, the association between frequent ED utilization and each SDoH or mental health risk factor was evaluated individually, adjusting for patient age, sex, race, ethnicity, primary language, means of arrival, arrival day of the week and time of day, chief complaint, triage ESI, MEWS24 score, age- and severity-weighted CCI score, insurance class, primary diagnosis, and ED disposition. Second, we assessed the mental health risk factors after adjusting for the presence of all other mental health risk factors along with the patient and visit characteristics. Third, the SDoH risk factors were assessed after adjusting for all mental health risk factors as well as patient and visit characteristics. Model results were reported as odds ratios (OR) with 95% confidence intervals (CI). We conducted all analyses using R v4.1.2 (R Foundation for Statistical Computing, Vienna, Austria).

### Data Source, Access and Cleaning

We retrieved all data from the electronic health record (Epic Systems Corporation, Verona, WI) used at each participating site. We extracted ED data from structured ED record sets, while SDoH survey data were obtained from ambulatory encounters in the same health system. We performed data extraction using Structured Query Language-based tools, linking the unique medical record numbers with the ED data and SDoH survey responses at the individual patient level. The process was automated and carried out by the biostatistician. No manual chart review was conducted.

### Missing Data

Visits with missing data from the SDoH survey were excluded from any analysis specific to the missing SDOH risk factors; visits with partial SDoH data were included for analysis on the available SDoH risk factors. Patients missing the ICD-10 codes flagged for mental health risk factors were considered to not have these risk factors present at the index visit. In all cases of missing data, the total number of patient visits included for analysis is provided.

## RESULTS

### Participant Enrollment

**Figure 1:**
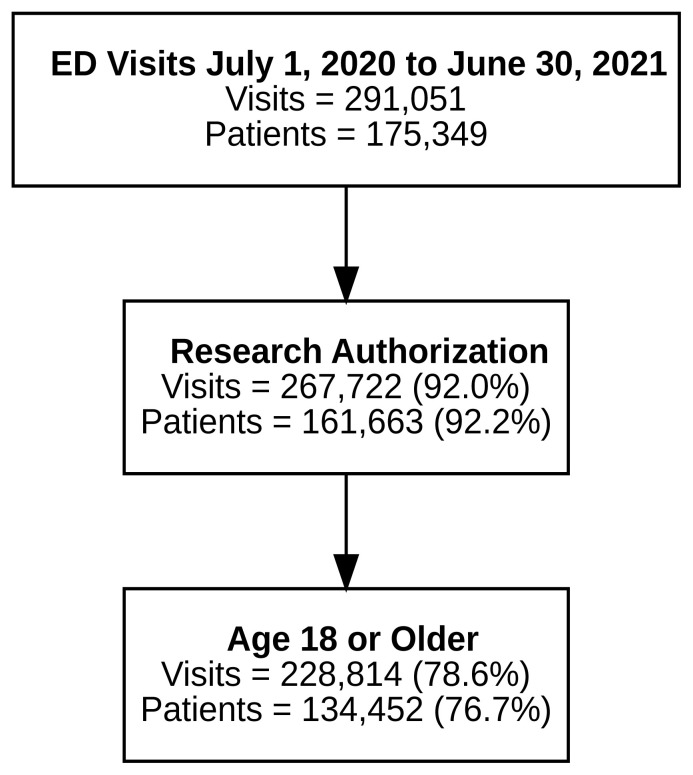
Participant enrollment in social determinants of health survey by number of visits and unique patients. *ED*, emergency department.

Participant enrollment in number of visits and unique patients is shown in Figure 1. We included patients ≥ 18 years of age who provided research authorization. Of the 228,814 visits included for analysis, 143,704 visits (79,597 distinct patients) (62.8%) had a response to at least one SDoH item, and 24,802 visits (13,691 distinct patients) (10.8%) had responses to all SDoH items. The reasons for non-participation pertained to institutional annual survey practices at primary care visits and access to primary care, as well as patient factors and choice in answering the survey questions.

### Patient Characteristics

The overall mean age for ED visits during the study period was 52.1 years, with 53.5% visits from females, 11.1% non-White, and 96.7% English-speaking. Insurance, means of arrival, CCI score, acuity level, and dispositions are included in Table 1. Primary mental health diagnoses included 1.2% for an alcohol-related diagnosis, 0.3% for a substance-related diagnosis, and 2.0% for a psychiatric-related diagnosis (Table 1). As compared with patients with ≤5 ED visits in the prior year, patients in the frequent-user cohort were more likely to be younger, female, and Black. They were also more likely to have Medicaid insurance, arrive by emergency medical services (EMS), have a higher CCI score, have higher proportions of digestive and psychiatric chief complaints, and ethanol-related visits. Patients with frequent ED use had death rates that were lower at seven days but higher at 90 days and 180 days (Table 1). All comparisons were significant, *P*< .001.

### Outcome Data

Overall, visits among patients with ≥3 previous ED visits accounted for 22.9% (52,299 / 228,814) of the total ED utilization, and visits among patients with ≥6 ED visits accounted for 9.0% (20,529/228,814) of total ED utilization (Table 2).

### Mental Health Disorder Associations with Frequent Emergency Department Use

**Figure 2:**
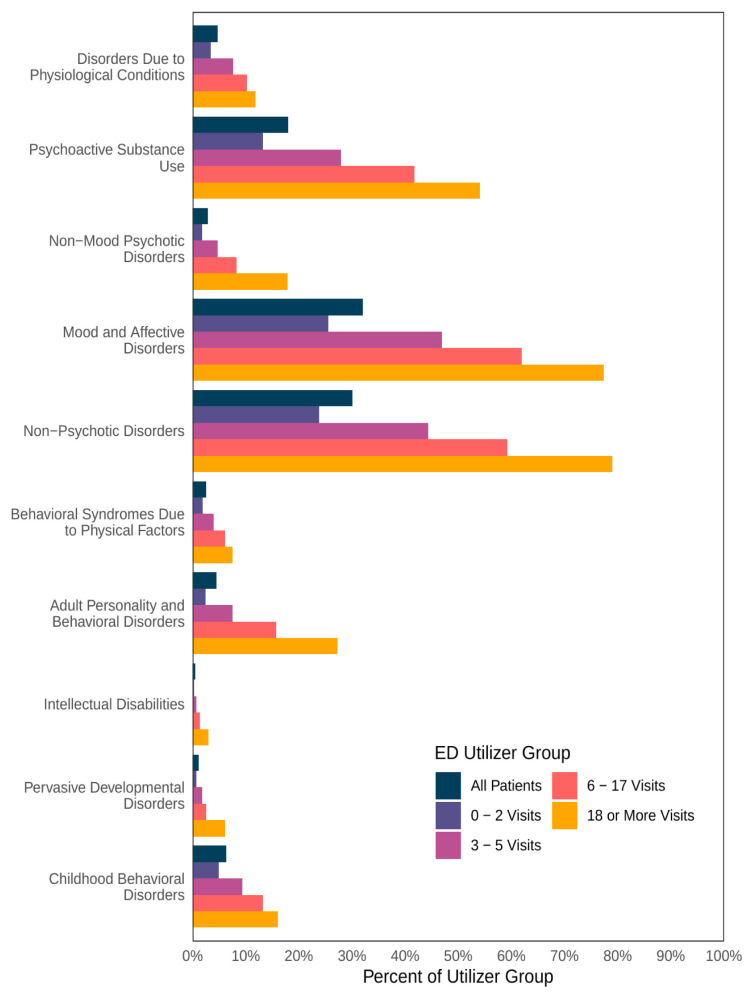
Mental health risk factors associated with emergency department use. *ED*, emergency department.

The most common ICD-10 mental health diagnosis codes were mood and affective disorders (32.0%), anxiety and other non-psychotic disorders (30.1%), and psychoactive substance use (17.9%). The patients with 6–17 visits had the same most common disorders but higher proportions of each (61.9%, 59.3%, and 41.8%, respectively) (Table 2). Mental health risk factors increased with frequency of ED utilization for all categories (Figure 2).

### Mental Health Risk Factors and Emergency Department Use

In a multivariable model adjusting for clinical features, all mental health diagnostic categories were significantly associated with frequent ED utilization as defined by having ≥6 annual ED visits, with the greatest odds of association with adult personality disorders (OR 4.00, 95% CI 3.79 – 4.23, *P* < .001), anxiety and other non-psychotic disorders (OR 3.35, 95% CI 3.23 – 3.47, *P* < .001), and mood/affective disorders (OR 3.23, 95% CI 3.11 – 3.34, *P* < .001). In the multivariable model adjusting for both clinical features as well as the presence of diagnoses in other mental health diagnostic categories, all categories of mental health diagnoses continued to be significantly associated with frequent ED utilization; however, the odds of association were attenuated. The diagnostic categories with the greatest associations were anxiety stress and other non-psychotic disorders (OR 1.95, 95% CI 1.87 – 2.03, *P* < .001), adult personality and behavior disorders (OR 1.91, 95% CI 1.80 – 2.02, *P* < .001), and non-mood psychotic disorders (OR 1.79, 95% CI 1.66 – 1.93, *P* < .001) (Table 3).

### SDoH and Mental Health Diagnoses Associations with Frequent Emergency Department Use

**Figure 3:**
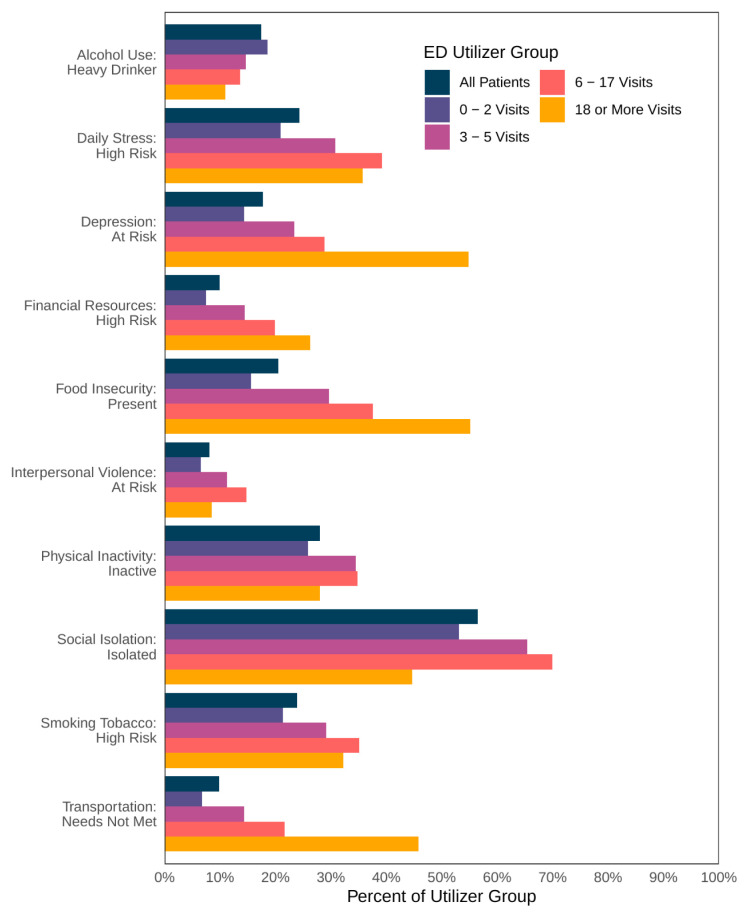
Social determinants of health risk factors associated with emergency department use. *ED*, emergency department.

The most common SDoH risk factors among the entire cohort included social isolation (56.5%), physical inactivity (28.3%), daily stress (24.3% high risk), and high-risk tobacco smoking (23.9%). For patients with 6 – 17 visits the most common SDoH risk factors were social isolation (70.0%), high-risk daily stress (39.3%), food insecurity (37.3%), and tobacco smoking (35.1% high risk). Patients with ≥ 18 ED visits had the highest proportions of depression risk (54.8%), food insecurity (55.1%), inadequate financial resources risk (26.3%), and unmet transportation needs (45.8%), but the lowest proportion of heavy alcohol use (10.9%) (Table 4). Item response rates ranged from 18.1 – 49.7% for the SDoH factors (Table 5). The SDoH risk factors increased with frequency of ED use for most categories with the exceptions of alcohol use and interpersonal violence, with tapering at the extremely high categories for daily stress, physical inactivity, social isolation, and tobacco use (Figure 3).

### Social Determinants of Health Risk Factors and Emergency Department Use

In a multivariable model adjusting for clinical features, most SDoH risk factors were significantly associated with frequent ED use (≥ 6 annual visits). Heavy alcohol use was associated with a decreased odds of high ED use (OR 0.72, 95% CI 0.66 – 0.79, *P* < .001) while the others were associated with an increased odds of frequent ED use. The greatest associations were for unmet transportation needs (OR 2.28, 95% CI 2.10 – 2.46, *P* < .001), inadequate financial resources risk (OR 2.04, 95% CI 1.88 – 2.20, *P* < 0.001), food insecurity (OR 2.00, 95% CI 1.88 – 2.14, *P* < .001), depression risk (OR 1.74, 95% CI 1.65 – 1.83, *P* < .001), and high risk of daily stress (OR 1.60, 95% CI 1.47 – 1.73, *P* < .001).

When adjusting for both clinical features and mental health diagnoses, the magnitude of most SDoH associations were attenuated and interpersonal violence, low risk for daily stress, and social isolation were not significantly associated with frequent ED use. The top three risk factors associated with the greatest odds of frequent ED use continued to be unmet transportation needs (OR 1.73, 95% CI 1.59–1.88, *P* < .001), food insecurity (OR 1.58, 95% CI 1.48–1.70, *P* < .001), and high risk for inadequate financial resources (OR 1.52, 95% CI 1.40–1.65, *P* < .001) (Table 5).

## DISCUSSION

This large multicenter study involving 228,814 patients across EDs in the Upper Midwest found significant associations between mental illness and SDoH with frequent ED use. This is in line with prior studies that demonstrate associations between increased healthcare utilization and mental health illness^1^,^5^,^6^,^7^,^8^,^13^–^15^ and SDOH risk factors such as homelessness.^1^,^10^,^14^,^15^ Our study encompasses a diverse range of settings, including academic, community, and critical access hospitals across two Midwestern states, broadening the perspective on frequent ED use.

We found that even after accounting for clinical and visit features and mental health risk factors, unmet transportation needs, high risk for financial resources, and food insecurity had the strongest associations with frequent ED utilization (Table 7). This adds to prior studies that focus on homelessness as a key risk factor,^1^,^8^,^11^ which may be highly correlated with financial strain, food insecurity, and transportation challenges. These findings are particularly pertinent to the population included in this study, which includes rural, geographically isolated EDs where patients may not have access to robust public transportation resources or strong social programs to address food insecurity and financial strain. The two mental health diagnostic categories most associated with frequent ED use were adult personality and behavioral disorders and anxiety, stress, and non-psychotic disorders. This is similar to prior research that demonstrated increased risk in conditions of depression, anxiety, personality disorders, and overall severe chronic mental health illness.^7^ After adjusting for all other mental health diagnostic categories there remained an almost doubling of the odds of frequent ED use for both (Table 7). The high prevalence of anxiety, stress, and non-psychotic disorders (30.1% of all visits, 61.7% 6+ visits) among frequent users is significant.

We observed a near doubling of the odds of anxiety and stressor personality and behavior disorders and an approximately 75% increased odds of psychotic disorders and mood disorders. The associations continue to be positive but attenuate after adjusting for clinical factors and other mental health diagnoses. We found that after accounting for clinical and visit characteristics and mental health risk factors, heavy alcohol use was associated with a 22% decreased odds of frequent ED use (Table 7). This is a novel finding not previously reported per our literature review and deserves further study. Prior studies show an association of alcohol and substance use with frequent ED use.^1^, ^3^, ^5^,^10^,^14^,^15^ Our findings could be driven by survey response biases in under-reporting alcohol consumption.

Similar to the findings of prior studies we found that ED frequent users are more likely to be younger, female, on Medicaid, arrive via EMS, have high comorbidity, and have digestive and psychiatric chief complaints.^27^ In a prior study of frequent ED use, mortality rates among frequent ED users ranged from no different to three times higher compared to non-frequent ED users.^18^ In our study we found that the odds of patient mortality were 17% lower, at seven days, and nearly 60% higher at 90 days and 180 days in the frequent-user group (≥6 ED annual visits compared to <6 visits in prior year) in line with the increased risk identified across other studies.

## LIMITATIONS

Our study has several limitations, the most significant being the methodology used to collect SDoH data. This information is gathered during outpatient visits within our organization, resulting in ≈60% of ED visits with at least one SDoH question completed, potentially affecting the generalizability to the broader ED cohort. For example, missing SDoH responses to sensitive or personal questions—potentially due to perceived stigma—may induce a sample bias leading to the under-representation of certain SDoH risk factors. However, with over 65,000 patients providing SDoH data, this still represents a substantial proportion of the total cohort, offering valuable insights that warrant further investigation. We hypothesize that SDoH factors will show even stronger associations when data is available for the entire ED patient population, although additional studies are needed to confirm this. In addition, our population is primarily White and speaks English as a primary language, which limits generalizability to more diverse populations outside Midwest rural populations. There may be unmeasured variables such as homelessness, which could result in confounding of results.

## CONCLUSION

We found strong associations between mental illness and social determinants of health and frequent ED utilization. The strongest SDoH risk factors include unmet transportation needs, financial resource risk, and food insecurity. These risks persist after adjusting for clinical features and mental health risk factors. Additionally, the top two mental health risk factors are adult personality and behavioral disorders as well as anxiety and stress, differences that persist when analyzed independently as well as when adjusting for other mental health risk factors.

Further understanding of the interaction between SDoH, mental health disorders, and frequent ED utilization, including in rural geographically isolated areas with limited access to public transportation and strong social programs, will empower care teams to address root causes and improve health outcomes among this vulnerable population. Further studies of rural healthcare systems could be compared to studies conducted in large metropolitan areas to better understand how interventions can be customized based on the needs of the population.

## Supplementary Information







## Figures and Tables

**Table 1:** Summary of patient demographics, visit characteristics, and outcomes

	All ED Visits (N = 228,814)	0–5 ED Visits in the Prior Year (N = 208,285)	6+ ED Visits in the prior year (N = 20,529)
Age, years			
Mean (SD)	52.1 (21.4)	52.4 (21.5)	49.5 (19.7)
Median (Q1, Q3)	52 (33, 70)	52 (33, 70)	48 (32, 64)
Sex, n (%)			
Female	122,415 (53.5%)	110,664 (53.1%)	11,751 (57.2%)
Male	106,392 (46.5%)	97,614 (46.9%)	8,778 (42.8%)
Unknown/did not disclose	7 (0.0%)	7 (0.0%)	0 (0%)
Race, n (%)			
American Indian/Alaskan Native	998 (0.4%)	853 (0.4%)	145 (0.7%)
Asian	2,771 (1.2%)	2,630 (1.3%)	141 (0.7%)
Black/African American	10,886 (4.8%)	9,442 (4.5%)	1,444 (7.0%)
Native Hawaiian/Pacific Islander	412 (0.2%)	373 (0.2%)	39 (0.2%)
White	203,339 (88.9%)	185,430 (89.0%)	17,909 (87.2%)
Mixed race	1,519 (0.7%)	1,216 (0.6%)	303 (1.5%)
Unknown/did not disclose	8,889 (3.9%)	8,341 (4.0%)	548 (2.7%)
Ethnicity, n (%)			
Not Hispanic or Latino	213,018 (93.1%)	193,642 (93.0%)	19,376 (94.4%)
Hispanic or Latino	11,476 (5.0%)	10,443 (5.0%)	1,033 (5.0%)
Unknown/did not disclose	4,320 (1.9%)	4,200 (2.0%)	120 (0.6%)
Primary language, n (%)			
English	221,226 (96.7%)	201,175 (96.6%)	20,051 (97.7%)
Spanish	2,948 (1.3%)	2,823 (1.4%)	125 (0.6%)
Somali	1,445 (0.6%)	1,314 (0.6%)	131 (0.6%)
Arabic	672 (0.3%)	597 (0.3%)	75 (0.4%)
Hmong	316 (0.1%)	278 (0.1%)	38 (0.2%)
Other language	1,648 (0.7%)	1,550 (0.7%)	98 (0.5%)
Unknown/did not disclose	559 (0.2%)	548 (0.3%)	11 (0.1%)
Language interpreter, n (%)			
No	147,930 (64.7%)	145,026 (69.6%)	2,904 (14.1%)
Yes	3,856 (1.7%)	3,810 (1.8%)	46 (0.2%)
Unknown/not recorded	77,028 (33.7%)	59,449 (28.5%)	17579 (85.6%)
Insurance type, n (%)			
Medicaid	51,544 (22.5%)	43,153 (20.7%)	8,391 (40.9%)
Medicare	85,769 (37.5%)	77,169 (37.0%)	8,600 (41.9%)
Other	10,658 (4.7%)	10,289 (4.9%)	369 (1.8%)
Private insurance	70,715 (30.9%)	67,981 (32.6%)	2,734 (13.3%)
Self-pay	10,124 (4.4%)	9,690 (4.7%)	434 (2.1%)
Unknown	4 (0.0%)	3 (0.0%)	1 (0.0%)
ED Region, n (%)			
MCHS Northwest Wisconsin	57,118 (25.0%)	51,849 (24.9%)	5,269 (25.7%)
MCHS Southeast Minnesota	51,105 (22.3%)	46,026 (22.1%)	5,079 (24.7%)
MCHS Southwest Minnesota	41,588 (18.2%)	37,665 (18.1%)	3,923 (19.1%)
MCHS Southwest Wisconsin	24,094 (10.5%)	22,001 (10.6%)	2,093 (10.2%)
Rochester	54,909 (24.0%)	50,744 (24.4%)	4,165 (20.3%)
Means of arrival, n (%)			
Non-EMS arrival	186,128 (81.3%)	170,838 (82.0%)	15,290 (74.5%)
EMS arrival	40,441 (17.7%)	35,391 (17.0%)	5,050 (24.6%)
Unknown	2,245 (1.0%)	2,056 (1.0%)	189 (0.9%)
Arrival Day of the Week, n (%)			
Sunday	30,756 (13.4%)	27,939 (13.4%)	2,817 (13.7%)
Monday	36,081 (15.8%)	32,874 (15.8%)	3,207 (15.6%)
Tuesday	33,353 (14.6%)	30,393 (14.6%)	2,960 (14.4%)
Wednesday	33,560 (14.7%)	30,595 (14.7%)	2,965 (14.4%)
Thursday	32,045 (14.0%)	29,090 (14.0%)	2,955 (14.4%)
Friday	32,502 (14.2%)	29,552 (14.2%)	2,950 (14.4%)
Saturday	30,517 (13.3%)	27,842 (13.4%)	2,675 (13.0%)
Arrival time of the day, n (%)			
Midnight to 6AM	20,819 (9.1%)	18,473 (8.9%)	2,346 (11.4%)
6am to Noon	64,659 (28.3%)	59,599 (28.6%)	5,060 (24.6%)
Noon to 6PM	86,914 (38.0%)	79,506 (38.2%)	7,408 (36.1%)
6PM to Midnight	56,422 (24.7%)	50,707 (24.3%)	5,715 (27.8%)
Triage ESI, n (%)			
Level 1	1,709 (0.7%)	1,575 (0.8%)	134 (0.7%)
Level 2	32,533 (14.2%)	29,113 (14.0%)	3,420 (16.7%)
Level 3	130,813 (57.2%)	119,064 (57.2%)	11,749 (57.2%)
Level 4	45,075 (19.7%)	41,605 (20.0%)	3,470 (16.9%)
Level 5	4,972 (2.2%)	4,499 (2.2%)	473 (2.3%)
Unknown	13,712 (6.0%)	12,429 (6.0%)	1,283 (6.2%)
MEW score, 0 – 14			
Mean (SD)	3.7 (1.0)	3.6 (1.0)	3.8 (1.0)
Median (Q1, Q3)	3 (3, 4)	3 (3, 4)	3 (3, 4)
Adjusted Charlson Comorbidity Index, 0 – 37			
Mean (SD)	4.0 (4.5)	3.8 (4.4)	5.8 (5.2)
Median (Q1, Q3)	2 (0, 7)	2 (0, 6)	4 (1, 10)
ED disposition, n (%)			
Discharge	168,043 (73.4%)	153,376 (73.6%)	14,667 (71.4%)
Admit	30,258 (13.2%)	27,685 (13.3%)	2,573 (12.5%)
Hospital observation	14,534 (6.4%)	13,117 (6.3%)	1,417 (6.9%)
Transfer – external	10,955 (4.8%)	9,790 (4.7%)	1,165 (5.7%)
Irregular departure	4,300 (1.9%)	3,656 (1.8%)	644 (3.1%)
Transfer – internal	151 (0.1%)	131 (0.1%)	20 (0.1%)
ED Observation	276 (0.1%)	251 (0.1%)	25 (0.1%)
Expired	195 (0.1%)	186 (0.1%)	9 (0.0%)
Unknown	102 (0.0%)	93 (0.0%)	9 (0.0%)
Chief complaint, n (%)			
Musculoskeletal	39,102 (17.1%)	36,122 (17.3%)	2,980 (14.5%)
Digestive	35,870 (15.7%)	32,101 (15.4%)	3,769 (18.4%)
General	33,857 (14.8%)	31,275 (15.0%)	2,582 (12.6%)
Respiratory	26,094 (11.4%)	23,679 (11.4%)	2,415 (11.8%)
Neurological	25,250 (11.0%)	22,910 (11.0%)	2,340 (11.4%)
Cardiovascular	21,414 (9.4%)	19,532 (9.4%)	1,882 (9.2%)
Skin	16,189 (7.1%)	15,285 (7.3%)	904 (4.4%)
Mental health	11,530 (5.0%)	9,661 (4.6%)	1,869 (9.1%)
Urological	6,101 (2.7%)	5,382 (2.6%)	719 (3.5%)
Eye	3,536 (1.5%)	3,386 (1.6%)	150 (0.7%)
Genital	2,127 (0.9%)	1,970 (0.9%)	157 (0.8%)
Ear	1887 (0.8%)	1,775 (0.9%)	112 (0.5%)
Pregnancy	1,087 (0.5%)	1,003 (0.5%)	84 (0.4%)
Endocrine	713 (0.3%)	614 (0.3%)	99 (0.5%)
Process	711 (0.3%)	585 (0.3%)	126 (0.6%)
Social problem	641 (0.3%)	584 (0.3%)	57 (0.3%)
Unknown	2,705 (1.2%)	2,421 (1.2%)	284 (1.4%)
Chief complaint – pain, n (%)			
Non-pain complaint	156,492 (68.4%)	142,826 (68.6%)	13,666 (66.6%)
Pain complaint	69,617 (30.4%)	63,038 (30.3%)	6,579 (32.0%)
Unknown	2,705 (1.2%)	2421 (1.2%)	284 (1.4%)
Primary mental health diagnosis, n (%)			
ETOH related	2,801 (1.2%)	2,188 (1.1%)	613 (3.0%)
Substance related	776 (0.3%)	649 (0.3%)	127 (0.6%)
Other psych diagnosis	4,504 (2.0%)	3,789 (1.8%)	715 (3.5%)
Other diagnosis	220,733 (96.5%)	20,1659 (96.8%)	19,074 (92.9%)
ED utilization in prior year, n (%)			
0 – 2 visits	176,515 (77.1%)	176,515 (84.7%)	0 (0.0%)
3 – 5 visits	31,770 (13.9%)	31,770 (15.3%)	0 (0.0%)
6 – 17 visits	17,977 (7.9%)	0 (0.0%)	17,977 (87.6%)
18 or more visits	2,552 (1.1%)	0 (0.0%)	2,552 (12.4%)
Patient mortality, n (%)			
Death within 7 days	1,697 (0.7%)	1,569 (0.8%)	128 (0.6%)
Death within 90 days	8,151 (3.6%)	7,079 (3.4%)	1,072 (5.2%)
Death within 180 days	11,787 (5.2%)	10,186 (4.9%)	1,601 (7.8%)

*ED*, emergency department; *SD*, standard deviation; *Q*, quartile.

*ED*, emergency department; *MCHS*, Mayo Clinic Health System; *EMS*, emergency medical services; *ESI*, Emergency Severity Index; *MEW*, modified early warning score; *SD*, standard deviation; *Q*, quartile.

*ED*, emergency department; *ETOH*, ethanol.

**Table 2:** Frequency of active mental health problems by ED utilization, n (%)

All patients	ICD-10 Codes	All visits (N = 22,8814)	ED utilization in the year prior to index visit			
			0 – 2 visits (N = 176,515)	3 – 5 visits (N = 31,770)	6 – 17 visits (N = 17,977)	18+ visits (N = 2,552)
Disorders due to physiological conditions	F01 – F10	10,643 (4.7%)	6,088 (3.4%)	2,425 (7.6%)	1,829 (10.2%)	301 (11.8%)
Psychoactive substance use	F11 – F19	41,060 (17.9%)	23,297 (13.2%)	8,875 (27.9%)	7,508 (41.8%)	1,380 (54.1%)
Non-mood psychotic disorders	F20 – F29	6,532 (2.9%)	3,119 (1.8%)	1,477 (4.6%)	1480 (8.2%)	456 (17.9%)
Mood/affective disorders	F30 – F39	73,168 (32.0%)	45,150 (25.6%)	14,916 (46.9%)	11128 (61.9%)	1,974 (77.4%)
Anxiety, stress and non-psychotic disorders	F40 – F49	68,873 (30.1%)	42,119 (23.9%)	14,079 (44.3%)	10,658 (59.3%)	2,017 (79.0%)
Behavioral syndromes due to physical factors	F50 – F59	5,809 (2.5%)	3,254 (1.8%)	1,267 (4.0%)	1,097 (6.1%)	191 (7.5%)
Adult personality and behavioral dsorders	F60 – F69	10,172 (4.4%)	4,282 (2.4%)	2,373 (7.5%)	2,821 (15.7%)	696 (27.3%)
Intellectual disabilities	F70 – F79	1,088 (0.5%)	532 (0.3%)	237 (0.7%)	242 (1.3%)	77 (3.0%)
Pervasive developmental disorders	F80 – F89	2,510 (1.1%)	1320 (0.7%)	576 (1.8%)	458 (2.5%)	156 (6.1%)
Childhood behavioral disorders	F90 – F98	14,482 (6.3%)	8,732 (4.9%)	2,966 (9.3%)	2,374 (13.2%)	410 (16.1%)

*ED*, emergency department.

**Table 3:** Association between active mental health diagnoses and frequent ED utilization

Mental health risk factor	Univariable^1^ 6+ ED visits		Multivariable^1^ 6+ ED visits	
	
	OR^2^ (95% CI)	P-value	OR^2^ (95% CI)	P-value
Disorders Due to Physiological Conditions	2.02 (1.91, 2.15)	< 0.001	1.31 (1.22, 1.39)	< .001
Psychoactive Substance Use	2.87 (2.76, 2.98)	< 0.001	1.72 (1.65, 1.79)	< .001
Non-Mood Psychotic Disorders	3.18 (2.97, 3.42)	< 0.001	1.79 (1.66, 1.93)	< .001
Mood/Affective Disorders	3.23 (3.11, 3.34)	< 0.001	1.73 (1.66, 1.81)	< .001
Anxiety, Stress and Non-Psychotic Disorders	3.35 (3.23, 3.47)	< 0.001	1.95 (1.87, 2.03)	< .001
Behavioral Syndromes Due to Physical Factors	2.32 (2.15, 2.50)	< 0.001	1.31 (1.21, 1.41)	< .001
Adult Personality and Behavior Disorders	4.00 (3.79, 4.23)	< 0.001	1.91 (1.80, 2.02)	< .001
Intellectual Disabilities	2.84 (2.42, 3.34)	< 0.001	1.56 (1.32, 1.85)	< .001
Pervasive Developmental Disorders	1.93 (1.73, 2.16)	< 0.001	1.23 (1.09, 1.39)	.001
Childhood Behavioral Disorders	1.88 (1.78, 1.99)	< 0.001	1.09 (1.03, 1.16)	< .01

1 Univariable models assess each mental health risk factor individually, multivariable models adjust for all other psych risk factors

2 Odds ratios were adjusted for patient age, sex, race, ethnicity, primary language, insurance, means of arrival, arrival day of the week and time of the day, ESI, MEW score, Charlson comorbidity, chief complaint, primary diagnosis, and ED disposition.

*ESI*, Emergency Severity Index; *MEW*, modified early warning score; *ED*, emergency department; *OR*, odds ratio.

**Table 4:** Frequency of SDOH risk factors by ED utilization, n (%)

Social determinant of health	Risk level	All visits	ED utilization in the year prior to index visit			
			0 – 2 visits	3 – 5 visits	6 – 17 visits	18+ visits
Alcohol use	Total responses	65,060	48,040	10,203	5,984	833
	Not at risk	53,743 (82.6)	39,128 (81.4)	8,704 (85.3)	5,169 (86.4)	742 (89.1)
	Heavy drinker	11,317 (17.4)	8,912 (18.6)	1,499 (14.7)	8,15 (13.6)	91 (10.9)
Daily stress	Total responses	74,373	55,007	11,666	6,779	921
	No risk	19,571 (26.3)	15,549 (28.3)	2,553 (21.9)	1,337 (19.7)	132 (14.3)
	Low risk	36,741 (49.4)	27,974 (50.9)	5,526 (47.4)	2,781 (41.0)	460 (49.9)
	High risk	18,061 (24.3)	11,484 (20.9)	3,587 (30.7)	2,661 (39.3)	329 (35.7)
Depression	Total responses	113,643	82,973	18,408	10,776	1,486
	Not at risk	93,499 (82.3)	71,057 (85.6)	14,098 (76.6)	7,673 (71.2)	671 (45.2)
	At risk	20,144 (17.7)	11,916 (14.4)	4,310 (23.4)	3,103 (28.8)	815 (54.8)
Financial resources	Total responses	68,919	50,630	10,925	6,505	859
	Low risk	49,709 (72.1)	39,038 (77.1)	6,823 (62.5)	3,557 (54.7)	291 (33.9)
	Medium risk	12,347 (17.9)	7,827 (15.5)	2,526 (23.1)	1,652 (25.4)	342 (39.8)
	High risk	6,863 (10.0)	3,765 (7.4)	1,576 (14.4)	1,296 (19.9)	226 (26.3)
Food insecurity	Total responses	62,972	45,939	10,077	6,089	867
	No insecurity	50,081 (79.5)	38,798 (84.5)	7,093 (70.4)	3,801 (62.4)	389 (44.9)
	Insecurity present	12,891 (20.5)	7,141 (15.5)	2,984 (29.6)	2,288 (37.6)	478 (55.1)
Inter-personal violence	Total responses	41,306	31,134	6,185	3,634	353
	Not at risk	37,988 (92.0)	29,081 (93.4)	5,486 (88.7)	3,098 (85.3)	323 (91.5)
	At risk	3,318 (8.0)	2,053 (6.6)	699 (11.3)	536 (14.7)	30 (8.5)
Physical inactivity	Total responses	68,084	50,614	10,594	6,198	678
	Sufficiently active	19,261 (28.3)	15,191 (30.0)	2,530 (23.9)	1,423 (23.0)	117 (17.3)
	Insufficiently active	29,756 (43.7)	22,356 (44.2)	4,414 (41.7)	2,615 (42.2)	371 (54.7)
	Inactive	19,067 (28.0)	13,067 (25.8)	3,650 (34.5)	2,160 (34.8)	190 (28.0)
Social isolation	Total responses	53,874	39,729	8,441	5,048	656
	Socially integrated	23,434 (43.5)	18,641 (46.9)	2,915 (34.5)	1,515 (30.0)	363 (55.3)
	Socially isolated	30,440 (56.5)	21,088 (53.1)	5,526 (65.5)	3,533 (70.0)	293 (44.7)
Smoking tobacco	Total responses	142,403	105,714	22,261	12,695	1,733
	Low risk	60,186 (42.3)	48,068 (45.5)	7,890 (35.4)	3,823 (30.1)	405 (23.4)
	Medium risk	48,170 (33.8)	35,109 (33.2)	7,872 (35.4)	4,420 (34.8)	769 (44.4)
	High risk	34,047 (23.9)	22,537 (21.3)	6,499 (29.2)	4,452 (35.1)	559 (32.3)
Transportation needs	Total responses	64,240	47,350	10,110	5,975	805
	Needs met	57,931 (90.2)	44,153 (93.2)	8,663 (85.7)	4,679 (78.3)	436 (54.2)
	Needs not met	6,309 (9.8)	3,197 (6.8)	1,447 (14.3)	1,296 (21.7)	369 (45.8)

*SDOH*, social determinants of health; *ED*, emergency department.

**Table 5:** Association between social determinants of health and frequent ED utilization

Social Determinant of Health		Adjusted for clinical features		Adjusted for clinical features and mental health risk factors	
	
		6+ ED Visits OR (95% CI)	P-value	6+ ED visits OR (95% CI)	P-Value
Alcohol use (N = 65,060)	Not at risk	-Reference-	---	-Reference-	---
	Heavy drinker	0.72 (0.66, 0.79)	< .001	0.78 (0.71, 0.85)	< 0.001
Daily stress (N = 74,373)	No risk	-Reference-	---	-Reference-	---
	Low risk	1.11 (1.03, 1.19)	.01	1.00 (0.91, 1.09)	.94
	High risk	1.60 (1.47, 1.73)	< .001	1.11 (1.03, 1.20)	.0
Depression (N = 113,643)	Not at risk	-Reference-	---	-Reference-	---
	At risk	1.74 (1.65, 1.83)	< .001	1.14 (1.08, 1.20)	< .001
Financial resources (N = 68,919)	Low risk	-Reference-	---	-Reference-	---
	Medium risk	1.61 (1.51, 1.73)	< .001	1.40 (1.30, 1.50)	< .001
	High risk	2.04 (1.88, 2.20)	< .001	1.52 (1.40, 1.65)	< .001
Food insecurity (N = 62,972)	No insecurity	-Reference-	---	-Reference-	---
	Insecurity present	2.00 (1.88, 2.14)	< .001	1.58 (1.48, 1.70)	< .001
Inter-personal violence (N = 41,306)	Not at risk	-Reference-	---	-Reference-	---
	At risk	1.44 (1.28, 1.62)	< .001	1.06 (0.94, 1.20)	.36
Physical inactivity (N = 68,084)	Sufficiently active	-Reference-	---	-Reference-	---
	Insufficiently active	1.19 (1.10, 1.28)	< .001	1.09 (1.01, 1.18)	.03
	Inactive	1.38 (1.27, 1.51)	< .001	1.23 (1.13, 1.34)	< .001
Social isolation (N = 53,874)	Socially integrated	-Reference-	---	-Reference-	---
	Socially isolated	1.06 (0.99, 1.14)	.09	0.98 (0.91, 1.05)	.55
Smoking tobacco (N = 142,403)	Low risk	-Reference-	---	-Reference-	---
	Medium risk	1.34 (1.27, 1.41)	< .001	1.24 (1.18, 1.31)	< .001
	High risk	1.58 (1.49, 1.66)	< .001	1.31 (1.24, 1.39)	< .001
Transportation needs (N = 64,240)	Needs met	-Reference-	---	-Reference-	---
	Needs not met	2.28 (2.10, 2.46)	< .001	1.73 (1.59, 1.88)	< .001

1 Clinical features include patient age, sex, race, ethnicity, primary language, insurance, means of arrival, arrival day of the week and time of the day, ESI, MEW score, Charlson comorbidity, chief complaint, primary diagnosis, and ED disposition.

*ED*, emergency department; *OR*, odds ratio; *CI*, confidence interval; *ESI*, Emergency Severity Index; *MEW*, modified early warning score.

**Table 6:** Odds of death for frequent utilizers relative to non-frequent utilizers

Odds of death	OR (95% CI)	P-Value
7 Days After ED Encounter	0.83, 95% CI: 0.69–0.99	p = .04
90 Days After ED Encounter	1.57, 95% CI: 1.47–1.68	p < 0.001
180 Days After ED Encounter	1.64, 95% CI: 1.56–1.74	p < 0.001

ED, emergency department; OR, odds ratio; CI, confidence interval.
