# Supplementary material for: Association of Mental Health Disorders and Social Determinants of Health with Frequent Emergency Department Use
Source: West J Emerg Med. 2025 Jul 18;26(4):905–17. doi: 10.5811/westjem.35599 (PMC12342428; doi:10.5811/westjem.35599)
Supplement: Supplementary file 1 [file wjem-26-905-g001.pdf]

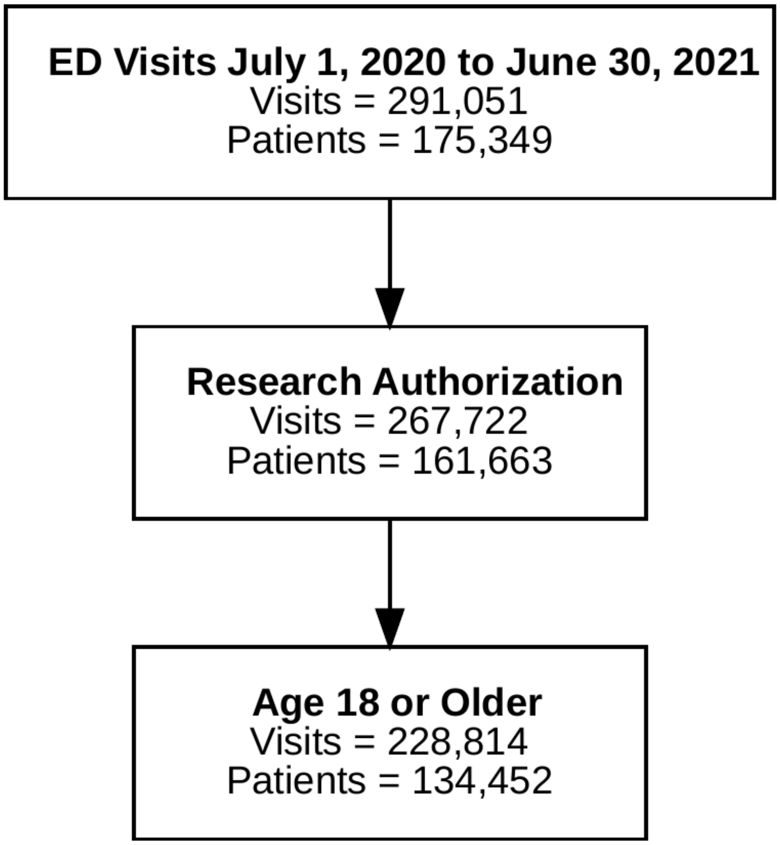
ED Visits July 1, 2020 to June 30, 2021
Visits = 291,051
Patients = 175,349
Research Authorization
Visits = 267,722
Patients = 161,663
Age 18 or Older
Visits = 228,814
Patients = 134,452
