# Supplementary figures and images for: Association of Mental Health Disorders and Social Determinants of Health with Frequent Emergency Department Use
Source: West J Emerg Med. 2025 Jul 18;26(4):905–17. doi: 10.5811/westjem.35599 (PMC12342428; doi:10.5811/westjem.35599)

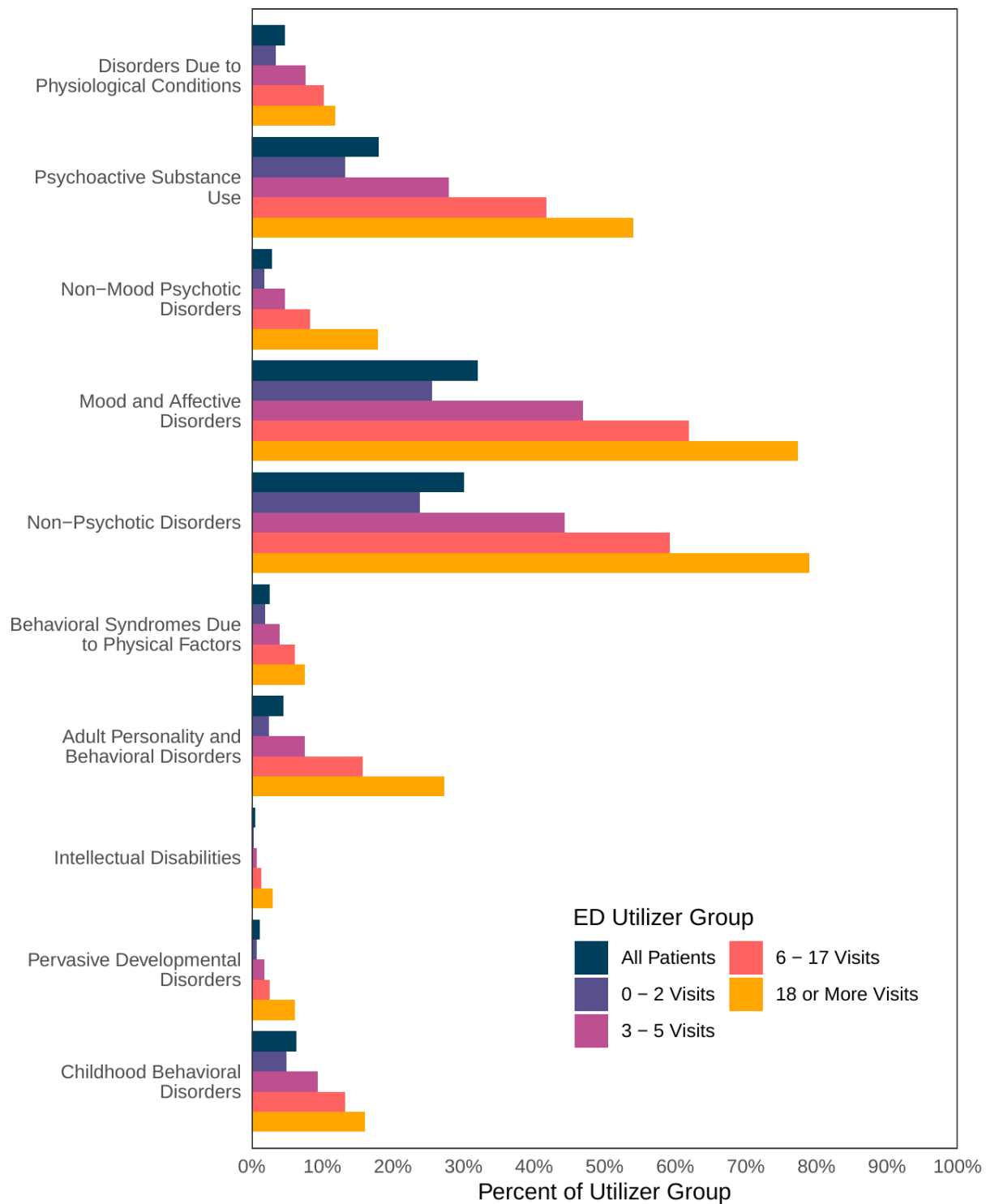

Supplement: Supplementary file 2 [file wjem-26-905-g002.pdf]

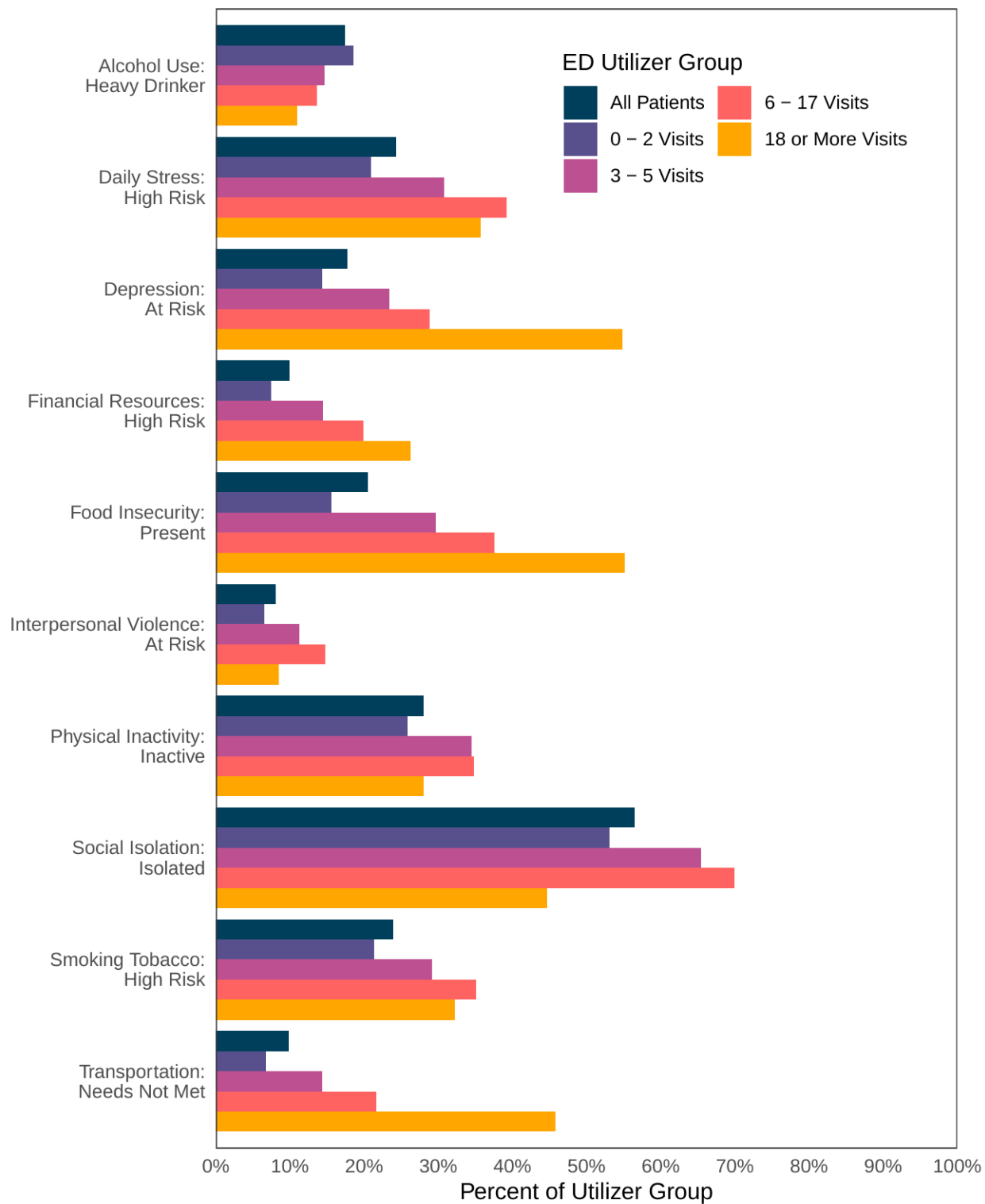

Supplement: Supplementary file 3 [file wjem-26-905-g003.pdf]
